# Wage Differentials between Heat-Exposure Risk and No Heat-Exposure Risk Groups

**DOI:** 10.3390/ijerph14070685

**Published:** 2017-06-24

**Authors:** Donghyun Kim, Up Lim

**Affiliations:** 1 Korea Environment Institute, 370 Sicheong-daero, Sejong 30147, Korea; donghyunkim@kei.re.kr; 2 Department of Urban Planning and Engineering, Yonsei University, 50 Yonsei-ro, Seodaemun-gu, Seoul 03722, Korea

**Keywords:** wage differential, heat-exposure risk, Blinder-Oaxaca method, Juhn-Murphy-Pierce method

## Abstract

The goal of this study is to investigate the wage differential between groups of workers who are exposed to heat and those who are not. Workers in the heat-exposure risk group are defined as workers who work in conditions that cause them to spend more than 25% of their work hours at high temperatures. To analyze the wage differential, the Blinder-Oaxaca and Juhn-Murphy-Pierce methods were applied to Korea Working Condition Survey data. The results show that the no heat-exposure risk group received higher wages. In most cases, this can be interpreted as the endowment effect of human capital. As a price effect that lowers the endowment effect, the compensating differential for the heat-exposure risk group was found to be 1%. Moreover, education level, work experience, and employment status counteracted the compensating differentials for heat-exposure risks. A comparison of data sets from 2011 and 2014 shows that the increasing wage gap between the two groups was not caused by systematic social discrimination factors. This study suggests that wage differential factors can be modified for thermal environmental risks that will change working conditions as the impact of climate change increases.

## 1. Introduction

Heat-exposure, which is predicted to occur as a result of climate change, has recently been discussed as a substantial change that can cause occupational risks in working conditions. The Intergovernmental Panel on Climate Change [1] forecasts that it is highly likely that within this century, the change in heat-exposure due to climate change will exceed the capacity of the human body to thermo-regulate in some regions and will impact the health and productivity of workers. The International Labor Organization (ILO) has also stated that climate change can lead to changes in working conditions and negatively impact vulnerable classes in the economy [2].

Previous studies on occupational risks have mainly discussed compensating wage differentials. Compensating differentials are expected wages that take occupational risks into consideration. A study in New York analyzing over 1600 jobs showed that expected wages increase by 1–4% per 10% increase in the variance of occupational risk [3]. Many studies that estimate the wage differentials for occupational risks operationalize the possibility of death and injury as the concept of risk, assess the level of compensating differentials in industrial sectors where occupational risks exist, and present wage–risk tradeoffs [4].

Of the many existing occupational risks and working conditions, the change brought about by heat-exposure is now more urgent because of the recent effects of climate change [5,6,7]. In relation to the occupational risks of heat-exposure, changes in a worker’s health conditions [5], as well as changes in productivity [8,9], have been presented. A worker’s heat-exposure risk refers to the level of exposure to working conditions that create heat stress during active work hours [7]. Workers who are exposed to extreme heat or work in hot environments indoors or outdoors, or even those engaged in strenuous physical activities, may be at risk of climate change impacts such as extreme heat exposure. With the increasing frequency and intensity of extremely hot weather predicted because of the changing climate, workplace heat exposure presents an increasing challenge to occupational health and safety. Extremely hot weather places many types of indoor and outdoor manual workers at an increasing risk of heat-related illnesses and injuries. In general, occupations with heat-exposure risks are considered to be dirty jobs and are more likely to be taken by people with a low education level and socioeconomic status [5,10,11,12].

Workers who risk exposure to heat are highly likely to manifest aspects of both social discrimination and compensating wage differentials for risks [13]. This implies that compensating differentials for heat-exposure risks affect the labor market, but can be offset by the variables of social discrimination. In other words, although workers who are exposed to occupational risks deserve to be fully compensated for these risks by the market, social inequality or discrimination can counterbalance the compensating wages for risks in the wage structure [3,14]. Furthermore, prolonged involuntary unemployment and economic recession can alter a worker’s choice and expected utility; it can also deteriorate the wage–risk tradeoff in conjunction with the company’s aversion to cost burden [13].

The goal of this study is to decompose the wage differentials between heat-exposure risk and no heat-exposure risk groups of workers to determine whether tradeoffs between compensating differentials and social discrimination exist and to investigate how these tradeoffs change over time. Data for this study were taken from the Korea Working Conditions Survey (2010, 2011, and 2014). To select the heat-exposure risk group, the amount of time exposed to heat during work hours was used as a key variable. To decompose wage differentials, this study created a wage determination model based on Mincer’s earnings function. The decomposition of wage differentials was conducted using the Blinder-Oaxaca method [15,16] and Juhn-Murphy-Pierce method [17]. The Blinder-Oaxaca method was utilized to divide the study subjects into the heat-exposure risk group and no heat-exposure risk group, and to determine whether the differentials were caused by an endowment effect (caused by compensating differentials) or by the price effect (caused by discrimination). The Juhn-Murphy-Pierce method was utilized to investigate the changes in wage differentials from 2011 to 2014.

## 2. Theoretical Discussion

### 2.1. Impacts of Heat-Exposure Risk on Labor

Climate change alters the working conditions related to workers’ risks. Changes to the thermal environment caused by climate change in turn cause changes to the working conditions, which pose occupational risks that future workers will face [7]. In addition, heat-exposure impacts worker productivity and occupational health [7,18,19]. Kjellstrom, Ingvar, and Bruno [8] forecasted a fall in work capacity using a biophysical model. There is a potential conflict between economic productivity and the health safety from heat stress [20]. For a worker, an increase in heat stress leads to an increase in time off work and decrease in work capacity [21]. In turn, the decreased work capacity leads to a decrease in productivity and earnings, which ultimately threatens the worker’s livelihood [22,23].

Some scholars have noted that the decline in labor productivity based on a human’s biophysical changes has already been observed during the hottest and most humid seasons in some regions of Asia and Africa [22,24], the Middle East [25,26], and Australia [27]. Moreover, even after greenhouse gases are reduced considerably, productivity is predicted to decrease to 20% by 2100 across the globe [24]. In particular, some regions in Southeast Asia are expected to withdraw from work for more than a half of the afternoon work hours in 2050 [28], and some occupation groups in South Korea are likely to experience the same phenomenon in 2050 [29]. Climate change issues related to workers are serious in developing countries in tropical zones; however, these issues are the same for workers with vulnerable occupations in advanced countries [7,30].

The decrease in labor productivity that could be caused by climate change may reinforce the discrimination against vulnerable social classes. Because a conflict exists between the impact on a worker’s health and productivity, the heat-exposure caused by climate change emerges as a critical risk to workers who are financially challenged and are given the minimum rights under harsh working conditions and strict work regulations [21]. Dangerous and difficult jobs are generally handled by the class of people who are socioeconomically discriminated against. Because they lack socioeconomic factors that increase earnings such as education, job skills, and experience, the compensating differential for dangerous work environments plays a major role in job selection. However, socially discriminated classes risk much more exposure to heat because of the impact of climate change [31,32], and pre-existing inequality increases and becomes chronic [1,33,34]. When a group vulnerable to social discrimination loses labor productivity due to any occupational risks from climate change, they may lose their basic financial capacity, which is their means of living [35]. After they lose their means of living, in addition to pre-existing discrimination, they may face a loss of savings, difficulties adjusting to changes in circumstances, and the trap of poverty, which ultimately lead to a prolonged state of poverty because they lack resilience to setbacks [1,36,37,38,39,40].

### 2.2. Compensating Differentials to Risk of Labor

Discussions on compensating differentials for worker occupational risks have long existed in the field of labor economics [41,42,43,44]. In the long-term equilibrium state of a market with market clearing, workers place themselves in the labor market by taking into account their personal preferences for occupational risks. For these risks, companies either pay the cost in the form of compensating wages or increase the marginal cost of their companies to create a safer working environment.

Natis, Michailidis, and Mattas [45] described the theoretical relationship between wage rates and risks. As the level of risk increases, the wage rate also rises, while the increase rate declines; this indicates an individual’s preference for risks and the wage–risk tradeoff. Workers who prefer low occupational risks have high risk compensation rates; in contrast, those who can withstand risks have low risk compensation rates. In other words, low-risk occupations have a relatively high level of compensation per risk unit [46].

When measuring the compensation level and the conflicting relationship between occupational risks and wages, researchers utilize models that take many variables into consideration. Most models primarily use the standard Mince earnings function [47], with risk as a main variable and the remaining variables as control variables that reflect individual characteristics. Polat [4] analyzed the risk premium of wages with a quantile approach based on the earnings distribution. Nastis, Michailidis, and Mattas [45] measured risk preferences by considering an individual’s behavior variables and estimated the conflicting relationship between wage rate and risk by representing them in the model. The research by Jacobs, Hartog, and Vijverberg [47] also included the variables of an individual’s risk aversion and preference, as well as the significance of the difference in an individual worker’s ability.

While the above study considered an individual worker’s ability and risk preference, Bender and Mridha [13] included employment structure variables. Bender and Mridha [13] suggested that a gap may exist in the conflicting relationship between risk and wages based on employment status. Whether an individual worker is actively employed or unemployed can change a company’s cost. In other words, involuntary unemployment lowers the expected utility for a worker’s risks, and the company may not pay the full cost for a worker’s safety. This is in line with Rosen’s finding [42] that the wage premium for risks can vary, depending on the labor market conditions.

Another aspect of this discussion concerns the value of a statistical life (VSL). To estimate the conflicting relationships between risk and wage, economists developed the concept of VSL [48]. Their intention was to connect risk with the mortality rate and estimate the increase in social welfare benefits due to the decline in premature deaths [49]. Since Schelling [50], VSL has been used to evaluate policies by determining how much benefit can be attained by an effort to reduce risk, while focusing on society’s willingness to pay for the effort or accept the risk based on the decline in mortality rate [48]. The model for measuring the VSL also used Mincer’s standard earnings function, with mortality rate as a variable for measuring the risks.

### 2.3. Wage Inequality of Labor

Various studies on the wage inequality of workers have measured the following wage differentials between groups with unequal conditions such as men and women [51,52], private and public sectors [53,54,55], different races [56,57], regional concentrations and characteristics [58,59], urban residents and migrant workers [60,61], and industry type [62]. These studies all utilized the wage function proposed by Mincer [63]. Mincer’s wage function consists of the number of years in education, number of years in the labor market, and their squares. When analyzing wage differentials, a revised wage function is used with additional explanatory variables suitable for the current research goals. After an earnings function appropriate for this study was created, the standard Blinder-Oaxaca wage differentials analysis [15,16] was used to investigate wage inequality. This method decomposes the variables into two parts: variables that can be explained by endowment and variables that cannot be explained by endowment and instead are affected by discrimination.

An extended analysis of the Blinder-Oaxaca standard method has recently been discussed. Depalo, Giordano, and Papapetrou [53] took the wage distribution into account and analyzed the gap between the private and public sectors. To investigate the effect of each group’s wage characteristics on the wage gap, they utilized a quantile decomposition analysis, which overcomes the necessity of discussing only average conditions when using the Blinder-Oaxaca method and allows each independent variable to contribute to the wage differential [64]. Juhn, Murphy, and Pierce [17] suggested taking several time units into account when applying the Blinder-Oaxaca method [58]. Chernozhukov, Fernandez-Val, and Melly [65] also proposed a quantile decomposition analysis approach. Shamsuddin [66] classified the findings of the Blinder-Oaxaca decomposition into four types and presented a strategy for identifying double discrimination.

As such, there are many studies that utilize extended approaches. When analyzing the wage differentials between the private and public sectors, Gürbüz and Polat [54] used not only the Blinder-Oaxaca method, but also an extended approach using the Juhn-Murphy-Pierce method [17] and the Chernozhukov, Fernandez-Val, and Melly method [65]. Kim, Min, and Choi [58] and Pereira & Galego [59] used the Juhn-Murphy-Pierce method to analyze the variables of wage differentials over time based on differences in the regional labor market. Piazzalunga [56] extended the standard Blinder-Oaxaca method and investigated the double negative effect. They first estimated the wage differentials based on race and gender using a pooled regression model, and then applied the Blinder-Oaxaca method. Following this, they redecomposed the Blinder-Oaxaca decomposition model using the Shamsuddin decomposition model [66], through which they postulated the double negative effect.

## 3. Methods

This study examines the form of wage differentials between the heat-exposure risk group and the no heat-exposure risk group to understand whether a tradeoff between compensating differentials and social discrimination exists within wage differentials and to investigate how these tradeoffs change over time. As mentioned in the literature review, an individual worker’s choice regarding risk leads to compensating wage differentials in the labor market. Nevertheless, a discussion on a reasonable level of compensating differentials ends with inconsistent conclusions; it implies that dangerous occupations or occupations with few amenities may have lower wages [13]. The causes include problems with wage measurement, measuring risks and endogeneity, the attraction to risk, and the structure of the labor market [13]. With this in mind, this study has been designed to investigate whether there are any wage differential variables based on heat-exposure risks. This study does not only postulate compensating differentials considered within the conflicting nature of wage and risks. The change in the thermal environment can create social discrimination variables, or there is the possibility of compensating differentials in the labor market. This study examines all of these possibilities as a gap created by unexplained factors.

In this study, a wage differential is defined as the difference in wages between the heat-exposure risk group and the no heat-exposure risk group. To investigate wage differentials, we should primarily analyze factors that influence wages. In this paper, we focus on individual workers’ human capital characteristics. Thus, we establish an empirical model that, based on human capital theory, incorporates the effect of productivity-related characteristics that represent the level of human capital on wages.

The wage differential between the heat-exposure risk and no heat-exposure risk groups are measured by the Blinder-Oaxaca method [15,16]. The Blinder-Oaxaca method can tell whether the differentials are based on the endowment effect, which consists of explainable elements such as an individual’s education level, experience, and occupation, or based on discrimination, which is not explainable. However, this method has the limitation that it shows the change in wage differentials over two years. This study uses the Juhn-Murphy-Pierce model [17] to investigate changes over time.

To understand the wage differential, the wage determinants could be considered using a wage equation model. This study employs the Mincerian wage equation model, which is the most widely used standard for empirical wage equations [67]. Jacob Mincer’s wage equation has for decades provided the canonical framework within which labor economists have modeled wage and estimated the returns of education. On the basis of both theoretical and empirical arguments, Mincer modeled the natural logarithm of wage as a function of years of education and years of potential labor market experience. It has provided the framework for understanding the relationship between wage and a host of other factors, including nonwage job characteristics. Workers who are exposed to extreme heat, perform prolonged work in hot environments, or are engaged in strenuous physical activity may be at risk of heat stress. In this study, workers in the heat-exposure risk group are defined as workers who work in conditions that cause them to spend more than 25% of their work hours at high temperatures. The characteristics of workplace heat exposure may vary in different occupations. The impacts of heat exposure can be particularly harsh on outdoor workers such as those in the agriculture, construction, mining, and manufacturing industries.

First, to identify the wage structure, a wage function based on the Mincerian wage equation [63] was used.

(1) $$lnw_{i}=\alpha_{i}+X_{i}\beta_{i}+\varepsilon_{i},$$

(2) $$W_{j}=lnw_{j}=X_{j}\beta_{j}+\varepsilon_{j},$$

(3) $$W_{k}=lnw_{k}=X_{k}\beta_{k}+\varepsilon_{k},$$

where the indices *i*, *j*, and *k* are for all individual workers in total, individuals who belong to the heat-exposure risk group, and individuals who belong to the no heat-exposure risk group, respectively. In Equations (1)–(3), α is a constant, w is the mean wage per hour, β is a coefficient of an independent variable, and X represents the vectors of observed individual characteristics that are assumed to determine the wage rate.

The Blinder-Oaxaca model is a well-known method for investigating labor market discrimination, and it has been applied to many variables including race and gender [68]. The goal of the Blinder-Oaxaca decomposition is to explain the difference in the average findings between two groups. In this study, the two groups are the heat-exposure risk group (*j*) and the no heat-exposure risk group (*k*). At one point in time *t*, the Blinder-Oaxaca decomposition is:

(4) $$W_{k}^{t}-W_{j}^{t}=\left(X_{k}^{t}-X_{j}^{t}\right)\hat{\beta_{k}^{t}}+X_{j}^{t}\;\left(\hat{\beta_{k}^{t}}-\hat{\beta_{j}^{t}}\right).$$

The left side of (4) refers to the difference in the log of the mean wage between the heat-exposure risk group and the no heat-exposure risk group at time *t*. Moreover, $X_{k}^{t}$ and $X_{j}^{t}$ are vectors of the mean observed characteristics and include other wage determinants such as human capital. Estimates $\hat{\beta_{k}^{t}}$ and $\hat{\beta_{j}^{t}}$ are ordinary least squares parameter estimates of the earning functions for groups *k* and *j*. The difference in the log of the mean wages is divided into two parts: the first part of the difference ($\left(X_{k}^{t}-X_{j}^{t}\right)\hat{\beta_{k}^{t}}$) can be measured by explanatory variables as characteristics of the endowment effect, and the other ($X_{j}^{t}\;\left(\hat{\beta_{k}^{t}}-\hat{\beta_{j}^{t}}\right))$ is the difference that cannot be explained by endowment. It is considered to be the price effect or the difference resulting from discrimination.

Juhn-Murphy-Pierce [17] extended the Blinder-Oaxaca decomposition model so that temporal change can be analyzed [69,70]. With regard to the unexplained part in (4), taking the group difference in the residuals into consideration leads to:

(5) $$D^{t}=W_{k}^{t}-W_{j}^{t}=\Delta X^{t}\hat{\beta_{k}^{t}}+\sigma_{k}^{t}\Delta \theta^{t}.$$

The decomposition of wage differentials taking into account the two time points, t′ and t, is:

(6) $$D^{t^{\prime }}-D^{t}=\left(\Delta X^{t^{\prime }}-\Delta X^{t}\right)\hat{\beta_{k}^{t^{\prime }}}+\Delta X^{t}\left(\hat{\beta_{k}^{t^{\prime }}}-\hat{\beta_{k}^{t}}\right)+\left(\Delta \theta^{t^{\prime }}-\Delta \theta^{t}\right)\sigma_{k}^{t^{\prime }}+\Delta \theta_{t}\;\left(\sigma_{k}^{t^{\prime }}-\sigma_{k}^{t}\right).$$

The first term on the right side in (6) is the observed *X*’s effect. This refers to the effect as a result of the change in an element observed through an explanatory variable in the model. This wage differential is created by the quantitative difference between the wage determinants of the two groups over time. The second term on the right side of (6) refers to the observed price effect. This is the manifested effect based on the change in compensation level in the market for the wage determinant or the element observed by the explanatory variable. The third term refers to the unobserved effect of *X* or the gap effect. This shows the relative change in position of the heat-exposure risk group within the wage distribution of the no heat-exposure risk group under the assumption that two groups have the same level of compensation. In other words, it estimates the effect of the differential that is not measured by explanatory variables over time [71]. The fourth term refers to the unobserved price effect; it indicates the degree of change in wage differential when the weighted value combined with the variable changes, while the differentials of the unmeasured properties between the two groups remain the same. It is the effect of the wage residual variance on wage differentials under the assumption that the mean wages of the two groups are stabilized after the observed characteristics of the explanatory variables are controlled for. This shows the impact of increased inequality throughout the society.

The variables used in this study are shown in Table 1. For wages, estimated hourly wages were used, reflecting the official inflation rates suggested by Statistics Korea. The elements of human capital include the number of years in education, number of years at work, and the square of the number of years at work, all of which are wage determinants. For the number of years in education, the rank variables were converted into the number of years in public education. The variables that impact the wage indirectly include the regional category (capital/non-capital region), type of employment (regular/non-regular), form of business (public/private), business size, gender, age, occupation, and industry category.

The present study used data from the Korea Working Condition Survey (KWCS) [72]. In this study, the measurement of heat exposure follows the criterion of KWCS, which is the average, comprehensive, and ordinary status of labor with respect to heat, not the exposure to heat due to the climatic conditions at a particular time. The first survey was conducted in 2006, and further surveys were conducted in 2010, 2011, and 2014. The KWCS [72] collected the data via one-to-one interviews with the working population over 15 years of age, using a sample survey from the Population and Housing Census of Korea. Because this study aimed to investigate the wage differentials, self-employed people and non-salary workers were excluded. Although data for four years was available, the first year data were excluded from this study because they were reported in the form of rank variables. When applying the Blinder-Oaxaca method, the data from the second, third, and fourth surveys were combined to obtain a higher number of samples. When applying the Juhn-Murphy-Pierce decomposition model, the third survey data set from 2011 was compared with the fourth survey data set from 2014 because the data size of the second survey from 2010 was small and unsuitable for comparison.

## 4. Results

### 4.1. Descriptive Statistics and Estimation of the Wage Function

The basic statistics of the wage determinants are shown in Table 2. According to the combined data from the second, third, and the fourth surveys, the hourly wage differential between the heat-exposure risk group and no heat-exposure risk group is about KRW 766.27 (USD 0.68). The wage differential from the third and fourth year surveys was KRW 661.99 (USD 0.59) and KRW 820.55 (USD 0.73), respectively. For the number of years in education, the no heat-exposure risk group had a higher mean; however, for the number of years at work, the heat-exposure risk group had a higher mean. The heat-exposure risk group was more concentrated in noncapital regions than the no heat-exposure risk group. In the no heat-exposure risk group, the number of regular employees was higher than that of non-regular employees. With regard to the gender ratio, the heat-exposure risk group had a higher male worker ratio; for average age, the heat-exposure risk group had a higher mean. With respect to the third year (2011), the overall wage decreased in the fourth year (2014). The degree of wage decrease in the heat-exposure risk group was greater than that of the no heat-exposure risk group, which led to even greater wage differentials. Other variables in the wage function did not demonstrate a substantial change. The education level slightly decreased in both groups, while work experience increased in both groups. Age also increased to a similar degree.

The estimated results of the wage function for use in the Blinder-Oaxaca and Juhn-Murphy-Pierce methods are shown in Table 3. The dependent variable is the log of hourly wages. In the model that includes both the heat-exposure risk and no heat-exposure risk groups, most variables were found to be statistically significant. The level of education and number of years at work were shown to have a positive impact on wage. In addition, working in the capital regions, being a regular employee, employment in the public sector, working in a large company, and being a male worker were found to have a positive impact on wage. The variables of heat-exposure risk had a positive impact on wage; however, the estimated result alone cannot determine what caused the positive impact. The no heat-exposure risk and heat-exposure risk groups also demonstrated a similar result, which was comparable to the general tendency.

### 4.2. Wage Differential Using the Blinder-Oaxaca Decomposition

The result of the Blinder-Oaxaca decomposition method is shown in Table 4 and Table 5. The wage differential between the no heat-exposure risk and heat-exposure risk groups was found to be about 11.3%. That is, the wage of the no heat-exposure risk group was 11.3% higher than that of the heat-exposure risk group. With respect to hourly wage, the difference between the groups was KRW 766 (USD 0.68). Considering that people work an average of eight hours per day and 20 days per month, the monthly wage differential is KRW 122,604 (USD 109.47), and the annual wage differential is estimated to be KRW 1,471,247 (USD 1313.61).

These wage differentials are generally explained by the wage determinant’s endowment effect and social factor’s price effect. In this study, the risk exposure group’s wage variables were set as low, but the interpretation of the data should take into account the fact that the wage premium for risk exists. Therefore, the wage determinant’s endowment effect refers to the wage differential based on the differences in wage determinants. In contrast, the price effect, which is the wage differential based on social factors, refers to the compensating differential for risks.

Overall, the coefficient (0.1237), which indicates the wage determinant’s endowment effect, is found to be higher than the wage differential (0.1132). This implies a vast wage differential because the no heat-exposure risk group experiences a greater endowment effect from the wage determinants. If the endowment effect of the wage determinants is applied to the no heat-exposure risk group at the level of endowment effect applicable to the heat-exposure risk group (the reference group of this study), the no heat-exposure risk determinants group would receive 12.3% higher wages than their counterparts. In other words, the reason for the heat-exposure risk group’s lower wages is found in the wage determinants such as education level, regular employment, and gender.

The unexplainable part, or discrimination factors, was found to reduce the gap caused by the endowment effect by 1.1%. Unlike the no heat-exposure risk group, the heat-exposure risk group receives wage premiums or a compensating differential for risks, and this differential is estimated to be around 1.1%. When all other working conditions are the same, no risk of exposure to heat means receiving a slightly lower wage. In other words, when a worker risks exposure to heat, the worker receives a higher wage for the heat-exposure risk; thus, it can be interpreted as wage premium for heat-exposure risks.

The variables of the wage differentials are shown in Table 5. Among the reasons for higher wages in the no heat-exposure risk group, the endowment effect wage determinants consist of the number of years in education, regular employment, and residency in the capital regions. In the no heat-exposure risk group, higher education levels have a stronger endowment effect on wage; among regular employees, employment in the capital regions displayed a stronger endowment effect. With regard to occupation type, professionals and related workers (Occ2) and elementary occupations (Occ9) exhibited a strong endowment effect. With regard to industry types, manufacturing (Ind3), construction (Ind6), accommodation and food service activities (Ind9), and business facility management and business support services (Ind14) manifested a stronger endowment effect.

In the heat-exposure risk group, the variables that display a strong endowment effect were gender and the number of years at work. This is because the main factors for high wages in the heat-exposure risk group are the male gender and the number of years at work. With regard to industry, a stronger endowment effect was manifested in the following categories: wholesale and retail trade (Ind7); financial and insurance activities (Ind11); education (Ind16); and human health and social work activities (Ind17).

Among the differences between the heat-exposure and no heat-exposure risk groups, a wage premium for the heat-exposure risk has a small explained part with very few determinants. In fact, the number of years in education, work experience, working in the capital regions, being a regular employee, and age were found to interfere with wage premiums. Occupation type makes a small contribution to wage premium; on the contrary, the opposite is true for industry type. It is difficult to interpret this as the pure effect of a wage premium because the constant term has some effect on dummy variables; however, the constant term displays a counteraction that impedes the wage premium.

### 4.3. Changes of Wage Differentials Using the Juhn-Murphy-Pierce Decomposition

Table 6 and Table 7, respectively show, the changes in the observed and unobserved effects derived from the Juhn-Murphy-Pierce approach. The log of the wage differential between the two times increases, and the change in the observed effect is 0.0292. In other words, the direction of the observed production element is positive, causing an increase in the differentials between the heat-exposure risk group and the no heat-exposure risk group. The observed price effect was negative, which indicates decreasing differentials between the heat-exposure risk group and no heat-exposure risk group.

The observed *X*’s effect is created by the quantitative differential of wage determinants that changes over time. In other words, it emerges with the change in the wage determinants of the heat-exposure risk and no heat-exposure risk groups. The sign of the value is positive, and the value is 0.0262. This is the wage differential resulting from the improvement of wage determinants in the no heat-exposure risk group. The sign of the observed price effect is negative, and this value is −0.0018. The change in income of the wage determinants over the period indicates that the income of the heat-exposure risk group increased slightly more than that of the no heat-exposure risk group. Nevertheless, the degree of change is not noticeable enough to counterbalance the observed *X*’s effect. The remaining change in the unobserved effect helps reduce the wage differential between the heat-exposure risk and no heat-exposure risk groups. However, the gap is very small and its impact is almost negligible. Such a change in the unobserved effect, unlike the change in the production factor, is caused by systematic or social discrimination factors. In this study, however, only a three-year time period is considered and such changes cannot be determined.

Table 8 shows the impact of the wage determinants that compose the observed *X*’s effect and the observed price effect. The details of the observed *X*’s effect on each wage determinant are as follows. Over time, the no heat-exposure risk group increased their education and number of years at work (or work experience) and workers employed in large companies increased their attributes, raising the wage in the no heat-exposure risk group. In addition, the endowment effect of the no heat-exposure risk group was distinctly manifested in workers in elementary occupations (Occ9), construction (Ind6), wholesale and retail trade (Ind7), accommodation and food service activities (Ind9), public administration and defense/compulsory social security (Ind15), and education (Ind16). In the heat-exposure risk group, the endowment effect was distinctly manifested in male workers and workers in the private sector. In the heat-exposure risk group, the endowment effect was large among plant or machine operator and assembler occupations (Occ8) and manufacturing (Ind3), and business facility management and business support services industries (Ind14).

With regard to price effects in the heat-exposure risk group, the temporal change in wage determinants was manifested in the increased education level and employment in the private sector. In the heat-exposure risk group, distinctive changes in the wage determinants were manifested in the occupations of professionals and related workers (Occ2), as well as the wholesale and retail trade (Ind7), financial and insurance activities (Ind11), education (Ind16), and human health and social work activities (Ind17) industries. In the no heat-exposure risk group, distinctive changes were manifested in the following occupation groups: regular employees, craft and related trade workers (Occ7), and elementary occupations (Occ9). Distinctive changes were also manifested in the following industry groups: agriculture, forestry, and fishing (Ind1), manufacturing (Ind3), construction (Ind6), accommodation and food service activities (Ind9), business facility management and business support services (Ind14), and membership organizations (Ind19). In contrast, with regard to the observed price effect, manufacturing and construction had the greatest impact, which implies improvement in the no heat-exposure risk group.

## 5. Conclusions

This study investigated the size of the wage gap between the group of workers that risk heat-exposure and the group of workers that do not risk heat-exposure. It was also determined how much of this gap is caused by social discrimination. The result of aggregating and analyzing three sets of KWCS data from 2010 to 2014 showed that the no heat-exposure risk group received higher wages; the differentials between the two groups were KRW 766 (USD 0.68) per hour, KRW 122,604 (USD 109.47) per month, and KRW 1,471,247 (USD 1313.61) per year. After decomposing the wage differential to determine whether this wage differential manifests the endowment effect of wage determinants, this study discovered that the wage premium for heat-exposure remained at approximately at 1%. This can be seen as a compensating differential, as suggested in previous theories. The reason for higher wages in the no heat-exposure risk group was explained using the endowment effect of a higher education level, regular employment, and working in the capital regions. However, the composition of the wage premium for risks or compensating differential interfered with wage determinants such as education, work experience, regular employment, age, and working in the capital regions. This implies that these factors inhibit workers’ reasonable compensation for risking exposure to heat in the labor market.

The comparison of two data sets from 2011 and 2014 to investigate the cause of increased wage differentials showed that most cases could be attributed to the endowment effect of wage determinants. The wage differential increased because the no heat-exposure risk group displayed a more distinctive improvement in the quantitative components of wage determinants over time. Although the marginal income of the wage determinant improved in the heat-exposure risk group, the amount was insignificant. In addition, while the gap effect, which indicates social discrimination, contributes to reducing the differential between the heat-exposure risk group and the no heat-exposure risk group, the analysis period and value in this study were too short and small, respectively, to determine this effect.

A change in the thermal environment can create social discrimination variables, or there is the possibility of compensating differentials in the labor market. The no heat-exposure risk group has a high level of human capital and an advantageous position in the labor market. In contrast, the heat-exposure risk group has insufficient human capital and a relatively disadvantaged position in the labor market. The polarization of the labor market is highly likely to divide the working conditions for such risks.

Climate change makes thermal extreme events like heatwaves more extreme and frequent, and it makes the future working conditions, in particular for occupations like outdoor workers, riskier with respect to the thermal environment [12]. In many cases, these workers are likely to be at a disadvantaged position in the labor market. Especially in a polarized labor market, extreme heat-related events due to climate change can add to the social discrimination of heat-exposure risk groups. Even if wage premiums for heat-exposure risks exist, the compensation is not significant enough to overcome the unequal conditions that pre-exist in the labor market. In particular, in labor markets without perfect competition, such risks are most likely to become a burden for workers in disadvantaged positions.

## Figures and Tables

**Table 1:** Variable definitions.

Variable	Definition	Measure
Ln_wage	Hourly wages	Reflects the official inflation rate by Statistics Korea (100 in 2010, 104 in 2011, and 109.04 in 2014), units: KRW 10,000 (USD 8.93), paid to workers only.
Edu	Number of years of schooling	No schooling or below elementary school = 3 years, elementary school graduates = 6 years, middle school graduates = 9 years, high school graduates = 12 years, junior college graduates = 14 years, four-year college graduates = 16 years, graduate school degrees = 18 years.
Exp	Number of years at work	Number of years employed at the current worksite.
Exp2	Square of the number of years at work	Square of the number of years employed at the current worksite.
Capital	Regional type	1 = capital regions, 0 = non-capital regions. Capital regions refer to Seoul, Gyeonggi, and the Incheon area. Non-capital regions refer to all other areas.
Regular	Type of employment	1 = regular employee, 0 = non-regular employee. Regular employees are workers whose employment contract is over a year. Irregular employees are temporary workers (1 month to 1 year) or daily laborers (under one month).
Private	Form of business	1 = private sector, 0 = public sector.
Large	Number of employees at the current worksite	1 = company with over 100 employees, 0 = company with under 100 employees.
Men	Gender	1 = men, 0 = women.
Age	Age	Exact age from birth.
Heat_risk	Heat-exposure risk	1 = exposure, 0 = non-exposure. Working conditions that expose workers to heat over 25% of the work hours (heat-exposure is defined by temperatures hot enough to make workers sweat even during their breaks).
Occ1–Occ9	Occupation dummies	Korean Standard Classification of Occupations: Occ1 = managers, Occ2 = professionals and related workers, Occ3 = clerks, Occ4 = service workers, Occ5 = sale workers, Occ6 = skilled agricultural, forestry, and fishery workers, Occ7 = craft and related trades workers, Occ8 = plant, machine operators and assemblers, Occ9 = elementary occupations (benchmark = armed forces).
Ind1–Ind20	Industry dummies	Korean Standard Industrial Classification: Ind1 = agriculture, forestry and fishing, Ind2 = mining and quarrying, Ind3 = manufacturing, Ind4 = electricity, gas, steam, and water supply, Ind5 = sewerage, waste management, materials recovery, and remediation activities, Ind6 = construction, Ind7 = wholesale and retail trade, Ind8 = transportation, Ind9 = accommodation and food service activities, Ind10 = information and communications, Ind11 = financial and insurance activities, Ind12 = real estate activities and renting and leasing, Ind13 = professional, scientific, and technical activities, Ind14 = business facilities management and business support services, Ind15 = public administration and defense or compulsory social security, Ind16 = education, Ind17 = human health and social work, Ind18 = arts, sports, and recreation related services, Ind19 = membership organizations, repair, and other personal services, Ind20 = activities of households as employers, undifferentiated goods- and services-producing activities of households for personal use (benchmark = activities of foreign organizations and bodies)

**Table 2:** Descriptive statistics.

Variable	Heat-Exposure (Years 2–4)		No Heat-Exposure (Years 2–4)		Heat-Exposure (Year 3)		No Heat-Exposure (Year 3)		Heat-Exposure (Year 4)		No Heat-Exposure (Year 4)	
	Mean	SD	Mean	SD	Mean	SD	Mean	SD	Mean	SD	Mean	SD
Ln_wage	−0.45	0.570	−0.33	0.560	−0.42	0.550	−0.32	0.540	−0.46	0.580	−0.34	0.570
Edu	12.06	2.815	13.60	2.673	12.18	2.659	13.68	2.565	11.97	2.942	13.57	2.743
Exp	8.60	8.155	7.29	6.989	8.22	7.822	6.86	6.710	9.18	8.556	7.91	7.284
Exp2	140.45	266.566	101.94	198.031	128.76	247.736	92.12	188.898	157.55	291.177	115.66	208.663
Capital	0.32	0.467	0.40	0.490	0.31	0.463	0.39	0.488	0.32	0.465	0.42	0.493
Regular	0.73	0.446	0.84	0.365	0.76	0.426	0.87	0.339	0.68	0.467	0.80	0.400
Private	0.89	0.309	0.84	0.364	0.91	0.279	0.85	0.360	0.88	0.331	0.84	0.364
Large	0.16	0.365	0.17	0.373	0.16	0.371	0.16	0.364	0.15	0.359	0.17	0.379
Men	0.68	0.465	0.53	0.499	0.73	0.444	0.57	0.494	0.63	0.483	0.47	0.499
Age	46.04	12.098	42.00	11.391	44.86	11.311	41.03	10.842	47.61	12.874	43.28	12.071
Occ1	0.02	0.124	0.02	0.132	0.02	0.148	0.02	0.143	0.00	0.065	0.01	0.096
Occ2	0.08	0.265	0.21	0.406	0.07	0.247	0.21	0.405	0.09	0.280	0.21	0.408
Occ3	0.08	0.264	0.28	0.448	0.08	0.266	0.28	0.449	0.08	0.267	0.28	0.451
Occ4	0.11	0.318	0.09	0.282	0.10	0.303	0.09	0.279	0.12	0.330	0.09	0.290
Occ5	0.06	0.245	0.14	0.345	0.06	0.232	0.13	0.341	0.08	0.268	0.14	0.351
Occ6	0.02	0.124	0.00	0.060	0.01	0.109	0.00	0.057	0.02	0.136	0.00	0.063
Occ7	0.21	0.411	0.06	0.243	0.24	0.428	0.07	0.256	0.19	0.389	0.05	0.221
Occ8	0.20	0.403	0.09	0.283	0.23	0.418	0.09	0.288	0.19	0.391	0.08	0.278
Occ9	0.22	0.412	0.11	0.317	0.20	0.397	0.11	0.308	0.23	0.422	0.12	0.321
Ind1	0.02	0.137	0.01	0.071	0.01	0.120	0.00	0.068	0.02	0.151	0.01	0.071
Ind2	0.00	0.046	0.00	0.023	0.00	0.049	0.00	0.023	0.00	0.022	0.00	0.023
Ind3	0.30	0.460	0.20	0.396	0.34	0.475	0.19	0.396	0.27	0.443	0.20	0.401
Ind4	0.01	0.078	0.01	0.083	0.01	0.088	0.01	0.093	0.00	0.062	0.00	0.063
Ind5	0.00	0.061	0.00	0.045	0.00	0.057	0.00	0.045	0.00	0.062	0.00	0.041
Ind6	0.14	0.343	0.05	0.219	0.12	0.328	0.05	0.217	0.14	0.350	0.05	0.216
Ind7	0.09	0.285	0.16	0.369	0.09	0.285	0.17	0.373	0.10	0.296	0.16	0.367
Ind8	0.04	0.204	0.04	0.199	0.05	0.211	0.04	0.205	0.04	0.195	0.04	0.188
Ind9	0.10	0.299	0.06	0.233	0.09	0.286	0.06	0.229	0.11	0.311	0.06	0.241
Ind10	0.01	0.103	0.03	0.161	0.01	0.111	0.03	0.166	0.01	0.088	0.02	0.152
Ind11	0.01	0.112	0.06	0.242	0.01	0.097	0.06	0.236	0.02	0.127	0.06	0.244
Ind12	0.02	0.152	0.03	0.164	0.02	0.144	0.03	0.163	0.03	0.164	0.03	0.168
Ind13	0.01	0.096	0.03	0.179	0.01	0.093	0.03	0.179	0.01	0.095	0.03	0.179
Ind14	0.06	0.240	0.04	0.201	0.06	0.242	0.04	0.184	0.06	0.246	0.05	0.216
Ind15	0.04	0.206	0.05	0.224	0.03	0.176	0.05	0.220	0.06	0.232	0.05	0.222
Ind16	0.04	0.196	0.10	0.300	0.04	0.188	0.11	0.311	0.04	0.197	0.09	0.282
Ind17	0.03	0.164	0.07	0.259	0.02	0.151	0.07	0.249	0.03	0.180	0.08	0.279
Ind18	0.01	0.091	0.01	0.100	0.01	0.087	0.01	0.106	0.01	0.084	0.01	0.087
Ind19	0.06	0.229	0.05	0.209	0.06	0.243	0.05	0.218	0.04	0.207	0.04	0.198
Ind20	0.00	0.053	0.00	0.070	0.00	0.049	0.00	0.054	0.00	0.058	0.01	0.089
*N*	9610		36,962		4563		19,594		4184		13,766	

**Table 3:** Estimated result of the wage function.

Variable	Pooled		No Heat-Exposure Risk Group		Heat-Exposure Risk Group	
Constant	−1.2235	(0.1986) ***	−1.2543	(0.2250) ***	−0.9251	(0.4208) *
Edu	0.0619	(0.0010) ***	0.0634	(0.0011) ***	0.0539	(0.0021) ***
Exp	0.0341	(0.0007) ***	0.0336	(0.0008) ***	0.0320	(0.0015) ***
Exp2	−0.0006	(0.0000) ***	−0.0005	(0.0000)***	−0.0006	(0.0000) ***
Capital	0.0703	(0.0038) ***	0.0821	(0.0041) ***	0.0184	(0.0092) **
Regular	0.1662	(0.0053) ***	0.1751	(0.0061) ***	0.1538	(0.0111) ***
Private	−0.0180	(0.0069) ***	−0.0167	(0.0073) **	−0.0176	(0.0197)
Large	0.1261	(0.0053) ***	0.1193	(0.0058) ***	0.1456	(0.0127) ***
Men	0.2086	(0.0042) ***	0.2064	(0.0045) ***	0.2041	(0.0111) ***
Age	0.0007	(0.0002) ***	0.0011	(0.0002) ***	−0.0012	(0.0005) ***
Occ1	0.2350	(0.0389) ***	0.2244	(0.0453) ***	0.1801	(0.0787) **
Occ2	0.1022	(0.0367) ***	0.0750	(0.0430) *	0.1230	(0.0727) *
Occ3	0.0383	(0.0363)	0.0133	(0.0426)	0.0299	(0.0718)
Occ4	−0.0885	(0.0369) **	−0.0979	(0.0434) **	−0.0938	(0.0720)
Occ5	−0.0278	(0.0370)	−0.0467	(0.0433)	−0.0482	(0.0740)
Occ6	−0.1748	(0.0459) ***	−0.1635	(0.0579) **	−0.1797	(0.0823) *
Occ7	0.0133	(0.0369)	−0.0174	(0.0435)	0.0431	(0.0717)
Occ8	−0.0418	(0.0368)	−0.0816	(0.0433) *	0.0114	(0.0719)
Occ9	−0.2378	(0.0368) ***	−0.2695	(0.0433) ***	−0.2051	(0.0715) ***
Ind1	−0.5972	(0.1960) ***	−0.5554	(0.2226) **	−0.7305	(0.4141) *
Ind2	−0.2884	(0.2041)	−0.2809	(0.2368)	−0.3763	(0.4223)
Ind3	−0.4120	(0.1945) **	−0.4026	(0.2202) *	−0.5093	(0.4121)
Ind4	−0.3729	(0.1956) *	−0.3665	(0.2213) *	−0.4640	(0.4156)
Ind5	−0.3289	(0.1979) *	−0.2967	(0.2245)	−0.4605	(0.4177)
Ind6	−0.4061	(0.1946) **	−0.4152	(0.2203) *	−0.4504	(0.4123)
Ind7	−0.4699	(0.1945) **	−0.4640	(0.2203) **	−0.5571	(0.4124)
Ind8	−0.4845	(0.1946) **	−0.4875	(0.2203) **	−0.5120	(0.4125)
Ind9	−0.4653	(0.1947) **	−0.4751	(0.2204) **	−0.5194	(0.4126)
Ind10	−0.4305	(0.1948) **	−0.4266	(0.2205) *	−0.5169	(0.4140)
Ind11	−0.2161	(0.1946)	−0.2215	(0.2203)	−0.1889	(0.4138)
Ind12	−0.6755	(0.1948) ***	−0.6666	(0.2205) ***	−0.7696	(0.4130) *
Ind13	−0.3922	(0.1947) **	−0.3916	(0.2204) *	−0.4348	(0.4144)
Ind14	−0.5754	(0.1947) ***	−0.5938	(0.2204) ***	-0.5908	(0.4125)
Ind15	−0.4939	(0.1945) **	−0.4795	(0.2202) **	−0.6378	(0.4130)
Ind16	−0.4252	(0.1945) **	−0.4156	(0.2202) *	−0.5523	(0.4130)
Ind17	−0.5242	(0.1946) ***	−0.5177	(0.2203) **	−0.6267	(0.4130)
Ind18	−0.4997	(0.1953) **	−0.5078	(0.2210) **	−0.5291	(0.4148)
Ind19	−0.4696	(0.1947) **	−0.4719	(0.2204) **	−0.5408	(0.4126)
Ind20	−0.4751	(0.1964) **	−0.4793	(0.2221) **	−0.4602	(0.4198)
Heat_risk	0.0105	(0.0048) **	—		—	
Adj. R-sq	0.5247		0.5382		0.4720	
*F*	1319.34		1134.78		227.07	
*N*	46,572		36,962		9610	

Notes: Standard errors are shown in parentheses. * *p*-value < 0.1, ** *p*-value < 0.05, *** *p*-value < 0.01.

**Table 4:** Overall result of the Blinder-Oaxaca decomposition.

Classification	Coefficient	
No heat-exposure risk group	−0.3342	(0.0029) ***
Heat-exposure risk group	−0.4474	(0.0058) ***
Wage differentials	0.1132	(0.0065) ***
Endowment effect	0.1237	(0.0051) ***
Price effect	−0.0105	(0.0051) **

Notes: Standard errors are shown in parentheses. ** *p*-value < 0.05, *** *p*-value < 0.01.

**Table 5:** Details of the Blinder-Oaxaca decomposition.

Variable	Endowment Effect		Price Effect	
Constant	—		−0.3292	(0.1171) ***
Edu	0.0955	(0.0026) ***	0.1172	(0.0333) ***
Exp	−0.0448	(0.0033) ***	0.0141	(0.0173)
Exp2	0.0234	(0.0021) ***	0.0113	(0.0090)
Capital	0.0054	(0.0005) ***	0.0214	(0.0035) ***
Regular	0.0192	(0.0011) ***	0.0165	(0.0110)
Private	0.0009	(0.0004) **	0.0008	(0.0196)
Large	0.0011	(0.0005) **	−0.0042	(0.0020) **
Men	−0.0316	(0.0013) ***	0.0019	(0.0081)
Age	−0.0028	(0.0010) ***	0.1079	(0.0284) ***
Occ1	0.0005	(0.0003)	0.0007	(0.0012)
Occ2	0.0135	(0.0037) ***	−0.0072	(0.0073)
Occ3	0.0078	(0.0055)	−0.0063	(0.0084)
Occ4	0.0023	(0.0008) ***	−0.0002	(0.0068)
Occ5	−0.0021	(0.0021)	−0.0013	(0.0056)
Occ6	0.0021	(0.0006) ***	0.0001	(0.0011)
Occ7	−0.0020	(0.0042)	−0.0083	(0.0112)
Occ8	0.0048	(0.0032)	−0.0143	(0.0111)
Occ9	0.0247	(0.0031) ***	−0.0107	(0.0121)
Ind1	0.0085	(0.0013) ***	0.0028	(0.0016) *
Ind2	0.0005	(0.0002) **	0.0002	(0.0002)
Ind3	0.0447	(0.0070) ***	0.0314	(0.0222)
Ind4	−0.0003	(0.0003)	0.0006	(0.0006)
Ind5	0.0006	(0.0003) **	0.0006	(0.0004)
Ind6	0.0347	(0.0055) ***	0.0056	(0.0095)
Ind7	−0.0343	(0.0048) ***	0.0088	(0.0085)
Ind8	0.0011	(0.0011)	0.0011	(0.0035)
Ind9	0.0194	(0.0030) ***	0.0048	(0.0074)
Ind10	−0.0068	(0.0011) ***	0.0010	(0.0012)
Ind11	−0.0107	(0.0031) ***	−0.0007	(0.0020)
Ind12	−0.0025	(0.0012) **	0.0025	(0.0021)
Ind13	−0.0094	(0.0016) ***	0.0004	(0.0012)
Ind14	0.0110	(0.0019) ***	0.0002	(0.0047)
Ind15	−0.0042	(0.0013) ***	0.0072	(0.0039) *
Ind16	−0.0256	(0.0039) ***	0.0060	(0.0044)
Ind17	−0.0235	(0.0030) ***	0.0033	(0.0031)
Ind18	−0.0008	(0.0005)	0.0002	(0.0009)
Ind19	0.0045	(0.0013) ***	0.0038	(0.0043)
Ind20	−0.0010	(0.0003) ***	−0.0001	(0.0005)

Notes: Standard errors are shown in parentheses. * *p*-value < 0.1, ** *p*-value < 0.05, *** *p*-value < 0.01.

**Table 6:** Juhn-Murphy-Pierce decomposition method (Stage 1).

Year	Wage Differentials in Log	Observed Effect	Unobserved Effect
2011	0.0961	0.1139	−0.0178
2014	0.1226	0.1431	−0.0205
Difference	0.0265	0.0292	−0.0027

**Table 7:** Juhn-Murphy-Pierce decomposition method (Stage 2).

**Change in the Observed Effect**	**Observed *X*’s Effect**	**Observed Price Effect**	**Interaction**
0.0292	0.0262	−0.0018	0.0048
**Change in the unobserved effect**	**Gap effect**	**Unobserved price effect**	**Interaction**
−0.0027	−0.0018	−0.0002	−0.0007

**Table 8:** Juhn-Murphy-Pierce decomposition of changes in wage differential.

Variable	Change in the Observed Effect	Observed *X*’s Effect (*Q*)	Observed Price Effect (*P*)	Interaction *Q* × *P*
Edu	0.0022	0.0066	−0.0041	−0.0003
Exp	0.0092	0.0030	0.0065	−0.0004
Exp2	−0.0038	0.0033	−0.0061	−0.0009
Capital	0.0021	0.0021	−0.0000	−0.0000
Regular	0.0084	0.0024	0.0052	0.0008
Private	−0.0044	−0.0017	−0.0057	0.0030
Large	0.0038	0.0032	−0.0002	0.0009
Men	−0.0000	−0.0016	0.0015	0.0001
Age	0.0000	−0.0007	0.0006	0.0001
Occ1	0.0018	0.0012	−0.0002	0.0009
Occ2	−0.0052	−0.0012	−0.0045	0.0005
Occ3	−0.0017	0.0000	−0.0017	−0.0000
Occ4	0.0023	0.0016	0.0003	0.0003
Occ5	−0.0010	0.0006	−0.0019	0.0003
Occ6	0.0005	0.0012	−0.0004	−0.0003
Occ7	0.0049	−0.0004	0.0068	−0.0015
Occ8	−0.0011	−0.0024	0.0017	−0.0004
Occ9	0.0117	0.0070	0.0036	0.0011
Ind1	0.0082	0.0024	0.0032	0.0026
Ind2	−0.0000	0.0000	0.0005	−0.0005
Ind3	0.0030	−0.0141	0.0377	−0.0207
Ind4	0.0001	0.0001	−0.0002	0.0001
Ind5	0.0007	0.0001	0.0004	0.0003
Ind6	0.0272	0.0038	0.0183	0.0052
Ind7	−0.0141	0.0032	−0.0212	0.0039
Ind8	0.0009	0.0001	0.0008	0.0001
Ind9	0.0154	0.0028	0.0092	0.0033
Ind10	−0.0044	0.0000	−0.0045	0.0000
Ind11	−0.0119	−0.0000	−0.0125	0.0006
Ind12	0.0018	0.0021	−0.0013	0.0010
Ind13	−0.0073	0.0001	−0.0075	0.0001
Ind14	−0.0022	−0.0048	0.0045	−0.0020
Ind15	0.0073	0.0062	−0.0042	0.0054
Ind16	−0.0059	0.0049	−0.0167	0.0059
Ind17	−0.0172	−0.0023	−0.0125	−0.0024
Ind18	0.0009	0.0010	−0.0004	0.0003
Ind19	−0.0011	−0.0021	0.0032	−0.0023
Ind20	−0.0019	−0.0015	−0.0000	−0.0003
Total	0.0292	0.0262	−0.0018	0.0048
